# Intussusception in a Premature Neonate: A Rare Often Misdiagnosed Cause of Intestinal Obstruction

**DOI:** 10.1155/2009/607989

**Published:** 2009-12-21

**Authors:** I. Loukas, N. Baltogiannis, C. Plataras, A.-V. Skiathitou, S. Siahanidou, G. Geroulanos

**Affiliations:** ^1^Department of Pediatric Surgery, “St. Sophia” Children's Hospital, 11527 Athens, Greece; ^2^Department of Pediatrics, Neonatal Unit, “St. Sophia” Children's Hospital, 11527 Athens, Greece

## Abstract

Intestinal obstruction in neonatal period is an emergency caused by many surgical causes. An extremely rare surgical cause in this group of age is intussusception which can be easily confused with other surgical entities. In several reports, a significant number of the infants who were included in the study population were believed to have necrotizing enterocolitis (NEC). We present a rare cause of small intestine obstruction in a preterm female infant that can be easily misdiagnosed and confused preoperatively with other clinical entities particular for this period.

## 1. Introduction

 Intestinal obstruction in neonatal period is one of the most common surgical emergencies and may be due to intrinsic developmental defects, abnormalities of peristalsis, or abnormal intestinal contents. Successful management depends on a timely accurate diagnosis and an effective therapy [1]. We present a case of a female neonate with ileoileal intussusception and the manner to prevent a misdiagnosis with NEC, two conditions that require different managements. 

## 2. Case Report

**Figure 1:**
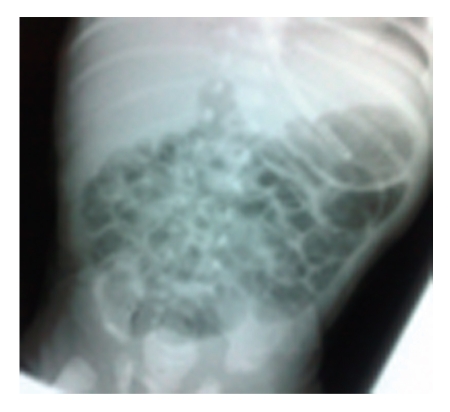
Abdominal radiograph that shows small intestine dilated loops in central position and a gasless distal part of intestine.

**Figure 2:**
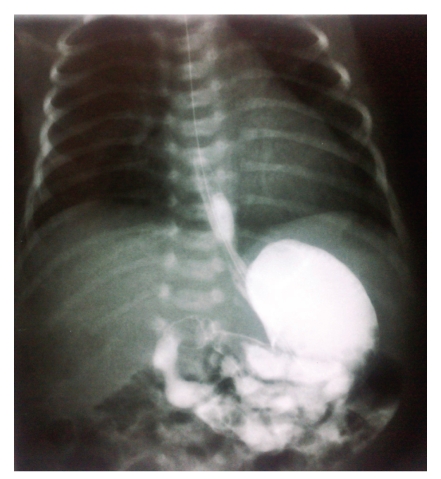
Upper gastrointestinal study with gastrografin shows an incomplete obstruction on jejunoileal junction.

A 2120 g female was delivered by cesarean section of 34 weeks because of a bigeminal gestation with an Apgar score of 8 in 1st minute and 9 in 5 minutes. The neonate passed meconium stools by the first hour after birth. Initially it was given parenteral feeding. Per os feeding was administrated on day 4 after birth. The neonate presented bilious vomiting and progressive abdominal distention on day 7. The feeding was progressively reduced and suppressed on day 9 due to bilious secretions from the nasogastric tube. Physical examination revealed a mild distended abdomen, hematological tests (WBC = 15300 /*μ*L, Hgb = 12.4 g/dL, Hct = 39%, PLT = 302.000 /*μ*L, CRP = 2.8, Glu = 97 mg/dL, Ure = 39 /mg/dL, Cre = 0.8 mg/dL, AST = 50 u/L, ALT = 42 u/L, Tbil = 1.63 mg/dL, Dbil = 0.53 mg/dL, K = 3.6 mmol/L, Na = 138 mmol/L, Cl = 102 mmol/L) ranged in normal with mild increasing of WBC and no bloody stools were presented. Antibiotics were given from the first day of life (3rd generation cephalosporines). On day 8 we performed an abdominal radiograph that demonstrated multiple air-filled bowel loops with mild dilatation of a few loops (Figure 1). Upper gastrointestinal study after the administration of gastrografin (on day 9) indicated a mild dilatation of the contrast filled loops and the presence of a significant amount of air, distal to the contrast filled loops that suggest an incomplete obstruction (Figure 2). The enema with gastrografin relieved a normal colon and ileocecal junction. On day 10 the child was operated on. The intraoperative findings were ileoileal intussusception, about 40 cm proximal to ileocecal junction, with moderate congestion of the intestinal wall of the 5 cm intussusceptum. The reduction was facile. No lead point was detected. The examination of the gastrointestinal tube proximal and distal is proved to be negative for other anomalies. The postoperative course was uneventful. The enteral feeding started at the 5th postoperative day and the child was discharged in an excellent general condition. 

## 3. Discussion

Intestinal obstruction in neonatal period may be due to duodenum or small intestinal atresia or stenosis, malrotation, meconium ileus, meconium plug syndrome, anorectal malformations, Hirschprung disease, ileus related to sepsis, and other rare causes [1]. 

Intussusception is the most common cause of intestinal obstruction at ages between 6–18 months but is an extremely rare clinical entity in neonates, especially among premature infants. It accounts for only 3% of intestinal obstruction and 0.3% (0%–2.7%) of all cases of intussusception [2]. In 370 cases of intussusception of our hospital in a 10-year period, we found only one case of neonatal intussusception (0.27%). Intussusception in infancy, childhood, and full-term neonates occurs most commonly at the level of the ileocolic junction. In all age groups, small bowel intussusception occurs in less than 10% yet is very common in premature neonates particularly in the ileum [2, 3]. Avansino et al. [4] in a review reported 35 cases of intussusception in premature infants. In 24 of these cases, it was localized in ileum. We found only 11 small bowel intussusceptions in 370 patients (3%). 

The etiology of neonatal intussusception in premature infants remains unclear. In full-term infants, it is associated with identifiable lead point [5]. In premature infants, it is suggested that common perinatal risk factors resulting in intestinal hypoperfusion/hypoxia, dysmotility, and stricture formation may act as a lead point for intussusception [5]. However, a lead point may be found in about 8% of cases. Ueki et al. [6] in a recent study of 14 neonates conclude that hypoxic events may play a crucial aetiologic role in the pathogenesis of late onset neonatal intussusception.

Intussusception occurring in premature neonates is difficult to assess. Typical clinical features are absent and the symptomatology is almost identical to NEC (abdominal distension, feeding intolerance, vomiting, and bloody stools). A palpable abdominal mass is present less commonly [2–7]. According to its high prevalence in this age group, NEC is the first think in these cases thus resulting in an average delay on the correct diagnosis of approximately 7 days [2, 7]. This average period gets longer in cases noncomplicated with perforation [7]. In our case the time between the examination of the child and the operation was 2 days. We used some clues to exclude the diagnosis of NEC such as the nondeterioration of the general condition, the presence of negative inflammation indicators especially CRP and PLT, the absence of both bloody stools and microscopically conditions that frequently occur in NEC, and finally, the radiograph findings discussed below. Indeed, Wang et al. [2] reported that intussusception must be highly suspected in a neonate who is diagnosed with NEC but who has a more stable course than would be expected.

Avansino et al. [4] mentioned that the most common imaging finding in patients with intussusception in premature neonates is dilated bowel loops. Similarly, in our case the radiograph of the abdomen relieved small intestine dilated loops. Pneumatosis intestinalis or portal venous gas has not been found (pathognomonic in about half of cases with NEC) [8]. Upper gastrointestinal study with gastrografin demonstrated an obstruction in the anatomical position of the jejunoileal junction, and the possible radiologic diagnosis given was this of type I intestinal atresia. The enema with gastrografin was normal. Due to these reasons, we did not performe abdominal ultrasonography. Martínez Biarge et al. [9] and Avansino et al. [4] in their recent studies concluded that ultrasound scan is capable to establish an early diagnosis of intussusception in neonates. With the experience gained with our case, we believe that a contrast enema is of a limited use because of the localization in ileum of intussusception at this age. The use of enemas should therefore be limited to positive and equivocal cases as well as to detect unsuspected disease. Indeed, ultrasound is a reliable imaging tool for rapid and accurate diagnosis. Advantages of ultrasound include the ability to document ileo-ileal intussusception, the absence of ionizing radiation to the neonate, and identification of lead points [9].

Intussusception in neonates being an extremely rare clinical entity will often be confused with other causes of intestinal obstruction and intestinal distention, and a high degree of suspicion may be needed to avoid the misdiagnosis. From all these conditions, it is very important to dissociate NEC, a condition which can be cured conservatively in most of the cases.
